# Effect and Relationship of Seasons on the High Risk of Ovarian Hyperstimulation Syndrome After Oocyte Retrieval in Patients With Polycystic Ovary Syndrome

**DOI:** 10.3389/fendo.2020.610828

**Published:** 2021-01-26

**Authors:** Yurong Cao, Hao Shi, Yue Ma, Linna Ma, Jun Zhai

**Affiliations:** ^1^Center for Reproductive Medicine, The First Affiliated Hospital of Zhengzhou University, Zhengzhou, China; ^2^Henan Key Laboratory of Reproduction and Genetics, The First Affiliated Hospital of Zhengzhou University, Zhengzhou, China; ^3^Henan Provincial Obstetrical and Gynecological Diseases (Reproductive Medicine) Clinical Research Center, The First Affiliated Hospital of Zhengzhou University, Zhengzhou, China

**Keywords:** polycystic ovary syndrome, ovarian hyperstimulation syndrome, temperature change, nomogram, receiver operating characteristic curve

## Abstract

**Objective:**

To investigate the effect of seasons on the incidence of high risk of ovarian hyperstimulation syndrome (OHSS) after in oocyte retrieval in patients with polycystic ovarian syndrome (PCOS) and to establish a nomogram to predict the risk of OHSS.

**Design:**

Single-center, retrospective study.

**Setting:**

University-affiliated reproductive medicine center.

**Patient(s):**

A total of 2,030 infertility patients with PCOS underwent the follicular phase long-acting long protocol IVF/ICSI in the reproductive medicine center from January 2017 to December 2019.

**Intervention(s):**

None.

**Main outcome measure(s):**

Logistic regression analysis was used to analyze the factors associated with a high risk of OHSS. We established a nomogram to predict the risk of OHSS in infertility patients with PCOS after oocyte retrieval.

**Result(s):**

The incidence of patients at high risk of OHSS was significantly different from season-to-season and was especially higher in the summer and winter. Multivariate logistic analysis showed that gonadotropin dosage, number of retrieved oocytes, estradiol level, average bilateral ovarian diameter on the day human chorionic gonadotropin was administered, type of infertility, and average temperature were independent risk factors for OHSS after oocyte retrieval in PCOS patients. Based on the above independent risk factors, we constructed a prediction model for OHSS risk. To evaluate the efficiency of the prediction model, we calculated the C-index (0.849), area under the receiver operating characteristic curve (0.849), and internal validation C-index (0.846). Decision curve analysis suggested that the prediction model exhibited significant net benefits.

**Conclusion(s):**

The incidence of PCOS patients at high risk for OHSS after oocyte retrieval fluctuated with seasonal temperature changes, and was significantly higher in extreme climates. The prediction model had favorable predictive performance and clinical application value.

## Introduction

Seasonal changes affect the development of many diseases and are closely related to the morbidity and mortality rate of hospitalization (1, 2). For instance, seasonal variations in acute coronary syndromes have been reported, with incidence and mortality peaking in the winter because of the proportion of plaque rupture is highest in winter (3). However, infectious and respiratory diseases are more prevalent in the summer (4).

Ovarian hyperstimulation syndrome (OHSS) is a severe complication of controlled ovarian hyperstimulation (COH) that is related to age, body mass index, ovarian function, and the ovulation stimulation protocol. The risk of OHSS in polycystic ovary syndrome (PCOS) patients is significantly increased (5). The etiology of OHSS is complex. Emerging evidence has shown that OHSS is associated with inflammatory factors interleukin (IL)-6, tumor necrosis factor (TNF)-α, IL-8, vascular endothelial growth factor, and the local renin-angiotensin aldosterone system, which led to a series of pathologic changes, including increased capillary permeability, leakage of vascular fluid into the interstitial space to form pleural effusions or ascites, decreased effective circulating blood volume, blood concentration, and even thrombosis (6, 7).

The immune system changes significantly throughout the year. Specifically, it has been shown that serum concentrations of IL-6 and soluble IL-6 receptor exhibit seasonality with higher expression during cold climates. IL-6 is an inflammatory factor that increases capillary permeability and plays an important role in the pathogenesis of OHSS (8, 9). Extreme weather activates the hypothalamic-pituitary-adrenocortical (HPA) axis and the sympathetic nervous system, which resulted in a high level of aldosterone (10). Abnormal expression of aldosterone promotes renal tubular reabsorption and increases the levels of inflammatory mediators, both of which play a key role in the occurrence of OHSS. The purpose of this study was to evaluate the relationship between the incidence of patients with PCOS at high risk for OHSS after oocyte retrieval and season and to construct a prediction model for OHSS risk to provide a new strategy to reduce the incidence of OHSS.

## Materials and Methods

### Study Design

A retrospective analysis of patients from Henan Province in China who were diagnosed with PCOS according to the Rotterdam criteria at The Center for Reproductive Medicine of The First Affiliated Hospital of Zhengzhou University, and underwent oocyte retrieval between 1 January 2017 and 31 December 2019. The follicular phase long-acting long protocol was used to stimulate follicles and *in vitro* fertilization (IVF)/intracytoplasmic sperm injection (ICSI) was performed. The study was retrospectively, the access and processing of patient data was approved by the ethics committee under a protocol for retrospective studies.

The inclusion criteria were as follows: 1) diagnosis of PCOS according to the Rotterdam criteria; 2) first fresh cycle using follicular phase long-term protocol and recived oocyte retrieval; and 3) age < 40 years. The exclusion criteria were as follows: 1) cycle cancellation of fresh embryo transfer because of abnormal liver function tests, high serum progesterone levels, pre-implantation genetic diagnosis/pre-implantation genetic screening, personal reasons, and/or uterine factors; 2) a history of OHSS or OHSS following embryo transfer; 3) a history of endometriosis, adenomyosis, surgery for ovarian cysts, hydrosalpinx, and pelvic tuberculosis; and 4) The male suffers from severe oligozoospermia or teratozoospermia.

Patients with PCOS at high risk for OHSS (serum estradiol [E2] level on human chorionic gonadotropin [HCG] administration day > 11,010 pmol/L, number of retrieved oocytes ≥ 15 and perceived bloating, and/or symptoms, such as bloating, abdominal pain, chest tightness, oliguria, pleural effusion, blood hypercoagulability, and/or volume of ovaries increased before retrieval) were required to cancel fresh embryo transfer and freeze embryos.

### Meteorological Data

Meteorological data for January 2017–December 2019 for Zhengzhou City, Henan Province, China were downloaded from the China Meteorological Data Net (http://data.cma.cn/) and included monthly minimum, maximum and average temperatures, and sunshine duration. Henan, China is located in the northern hemisphere with a warm temperate–subtropical monsoon climate. The dates of oocyte retrieval were divided into spring (March–May), summer (June–August), autumn (September–November), and winter (December–February).

### Ovulation Stimulation Program and Embryo Transfer

On the 2^nd^–3^rd^ days of menstruation, patients were given a long-acting gonadotropin releasing hormone agonist (Diphereline, 3.75 mg; Beaufour-Ipsen, Dreux, France) by subcutaneous injections. Thirty days later, we obtained blood from patients to determine serum follicle-stimulating hormone (FSH), luteinizing hormone (LH), E2, and progesterone (P) levels. At the same time, vaginal ultrasound was used to monitor the size of antral follicles. When the FSH level was < 5 IU/L, the LH level was < 3 IU/L, and the antral follicle was nearly 5 mm in diameter, COH was initiated. Based on patient age, anti-Mullerian hormone level, antral follicle count, body mass index, and serum FSH level, we determined the individualized dosage of gonadotropin ([Gn] GONAL-f; Merck Serono, Darmstadt, Germany). When one dominant follicle was ≥ 20 mm in diameter and at least three dominant follicles were ≥ 17 mm in diameter, a trigger injection of HCG (recombinant human chorionic gonadotropin alfa for injection, Merck Serono) was administered the same night. After 36–37 h of the trigger injection, we performed transvaginal oocyte retrieval. The method of fertilization depended on semen quality. Fresh embryo transfer was performed 3–5 days after oocyte retrieval based on embryo quality, endometrial and patient’s condition. The transplant was cancelled if patients were deemed at high risk for OHSS, the P level was > 3 ng/ml, or a uterine effusion was demonstrated.

Our primary outcome measure was the incidence of patients with PCOS at high risk for OHSS, calculated as the number of patients at high risk for OHSS per total number of patients. Secondary outcome measures were as follows. The fertilization rate was defined as the total number of fertilization oocytes per total number of retrieved oocytes. The cleavage rate was calculated as the total number of cleavages divided by the total number of fertilization oocytes. The high-quality embryo rate is expressed as the total number of high-quality embryos divided by the total number of cleavages. The clinical pregnancy rate was defined by the presence of a fetal heartbeat at 6–7 weeks of pregnancy. The live birth rate was defined as the total number of women with live births per total number of women with fresh embryo transfer.

### Statistical Analysis

For comparison of continuous variables between multiple groups and when the variance was homogeneous among groups, one-way ANOVA or the Kruskal-Wallis non-parametric test was used. The LSD-t test was used for a pairwise comparison of continuous variables within the group, and a chi-square test was used for comparison of proportions (Bonferroni correction was used to account for multiple testing). Linear regression analysis was used when the outcomes were continuous variables. Multivariable logistic regression analysis was used when the outcomes were dichotomous variables. The features were considered as odds ratios (ORs) with 95% confidence intervals (CIs) and a P-value. According to the regression coefficient of the final variable, a personalized prediction model was constructed. All potential predictors were applied to develop the prediction model. To evaluate the accuracy and differentiation of the prediction model, the C-index and receiver operating characteristic curve (ROC) were measured. The prediction model was subjected to bootstrapping validation (1000 bootstrap resamples) to calculate a relatively corrected C-index. Decision curve analysis (DCA) was performed to determine the clinical usefulness of the prediction model by quantifying the net benefits at different threshold probabilities in the cohort. A *P* ≤ 0.05 was considered statistically significant. Data analysis was conducted using SPSS 26.0 (Armonk, New York, USA) and R software (version 3.6.0; Miami, FL, USA). Delete sample objects with missing values under indicators.

## Results

**Figure 1:**
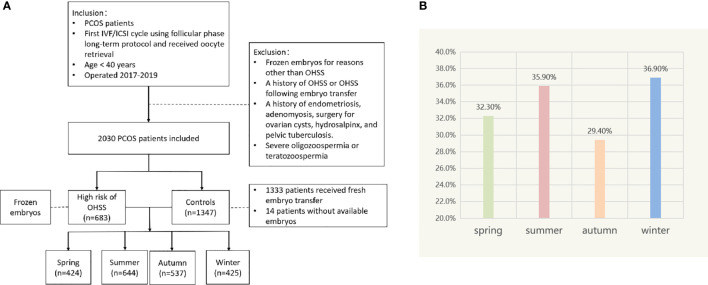
**(A)** Flow chart depicting the patient selection. **(B)** Incidence of patients at high risk of OHSS in different seasons.

A total of 2,030 patients with PCOS were included. Among them, 683 women with the high risk of OHSS cancelled fresh embryo transfer and frozen embryos, and the rest of the women served as controls, including 1,333 women who received fresh embryo transfer and 14 women without available embryos. We divided patients into four groups by season and compared various indicators of them. The results indicated that the number of retrieved oocytes, average bilateral ovarian diameter on the day of HCG administration, Gn dosage, incidence of patients at high risk for OHSS, and live birth rate were statistically different between seasons (*P* < 0.05; **Table 1** and **Figure 1**).

**Table 1:** Baseline characteristics and cycle outcomes in different seasons.

	Spring (Mar-May)	Summer (Jun-Aug)	Autumn (Sept-Nov)	Winter (Dec-Feb)	F	*P* value
Age, y	28.44 ± 3.69	28.8 ± 3.58	28.87 ± 3.64	28.91 ± 3.85	1.47	0.220
Fertilization method					—	0.382
IVF	85.8%(364/424)	86.9%(560/644)	83.4%(448/537)	85.8%(365/425)	—	
ICSI	14.2%(60/424)	13.1%(84/644)	16.6%(89/537)	14.2%(60/425)	—	
Type of infertility						0.109
Primary infertility	38.2%(162/424)	37.7%(243/644)	32.6%(175/537)	39.5%(168/425)	—	
Secondary infertility	61.8%(262/424)	62.3%(401/644)	67.4%(362/537)	60.5%(257/425)	—	
Infertility duration, y	3.9 ± 2.42	4.19 ± 2.76	4.11 ± 2.91	3.92 ± 2.57	1.38	0.247
BMI, kg/m2	24.39 ± 3.45	24.24 ± 3.21	24.48 ± 3.38	24.21 ± 3.39	0.73	0.532
Basal serum FSH level, U/L	5.85 ± 1.65	5.71 ± 1.5	5.92 ± 1.55	5.81 ± 1.65	1.83	0.139
Basal serum E2 level, pmol/L	46.24 ± 45.72	48.53 ± 40.88	52.80 ± 49.85	47.37 ± 41.20	0.152	0.118
Basal serum LH level, U/L	10.12 ± 7	10.29 ± 7.3	10.06 ± 7.87	9.99 ± 8.94	0.15	0.932
Basal serum AMH level, ng/ml	8.08 ± 4.25	8.46 ± 4.44	8.17 ± 4.27	8.38 ± 4.23	0.85	0.469
AFC	21.87 ± 5.43	22.17 ± 4.86	22.47 ± 4.64	22.2 ± 4.75	—	0.790
Number of retrieved oocytes	18.74 ± 7.91	17.65 ± 7.49^cd^	18.9 ± 7.97	19.13 ± 8.1	4.05	0.007
Endometrial thickness, mm	12.4 ± 2.62	12.195 ± 2.33	12.029 ± 2.53	12.287 ± 2.41	—	0.160
Serum E2 level on HCG day, pmol/L	1,059.88	1,006.95	962.95	1,015.23	—	0.086
Serum P level on HCG day, nmol/L	0.88 ± 0.58	0.81 ± 0.54	0.83 ± 0.51	0.84 ± 0.54	1.14	0.333
Average size of bilateral ovaries on HCG day, cm	5.44 ± 1.03	5.28 ± 0.99^d^	5.31 ± 0.93	5.45 ± 1.04	3.81	0.010
Gn dosage, U	2165.18 ± 914.91	2,082.16 ± 802.94^cd^	2,220.9 ± 878.52	2,253.56 ± 921.4	4.08	0.007
Incidence of high risk of OHSS	32.3%(137/424)	35.9%(231/644)	29.4%(158/537)	36.9%(157/425)	—	0.044
Fertilization rate	61.7%(4,903/7,947)	60.2%(6,839/11,364)	60.8%(6,166/10147)	60.7%(4,936/8,129)	—	0.211
Cleavage rate	98.9%(4,849/4,903)	98.7%(6,749/6,839)	98.8%(6,090/6,166)	98.9%(4,881/4,936)	—	0.675
High-quality embryo rate	60.2%^cd^(2,917/4,849)	60.5%^cd^(4,084/6,749)	64.4%^ab^(3,920/6,090)	63.3%^ab^(3,088/4,881)	—	0.000
Clinical pregnancy rate	76.0%(215/283)	76.7%(310/404)	74.5%(281/377)	74.7%(201/269)	—	0.887
Live birth rate	66.43%(188/283)	68.07%^c^(275/404)	58.62%(221/377)	62.45%(168/269)	—	0.035

BMI, body mass index; AFC, antral follicle count; OHSS, ovarian hyperstimulation syndrome; FSH, follicle stimulatine hormone; LH, luteinizing hormone; AMH, anti-mullerian hormone; E2, estradiol; P, progesterone^a^Significantly different from spring.

^a^Significantly different from spring ^b^Significantly different from summer ^c^Significantly different from autumn ^d^Significantly different from winter.

We analyzed the impact of season changes on the occurrence of patients at high risk for OHSS. Logistic regression analysis showed that Gn dosage, number of retrieved oocytes, serum E2 level, average bilateral ovarian diameter on the day of HCG administration, etiology of infertility, average temperature, average minimum temperature, and average maximum temperature were independent risk factors affecting OHSS risk in patients with PCOS (*P* < 0.05; **Table 2**).

**Table 2:** Association of high risk of ovarian hyperstimulation syndrome (OHSS) with meteorological factors.

	*P* value	OR	95% CI	
			Lower limit	Upper limit
Average temperatures, °C	0.003	0.395	0.214	0.727
Average minimum temperatures, °C	0.016	1.416	1.066	1.882
Average maximum temperature, °C	0.002	1.843	1.256	2.704
Sunshine duration, hr	0.153	0.996	0.991	1.001

OR, odd ratio; 95% CI, 95% confidence interval.

Calibration variables: age, infertility duration, BMI, basal serum FSH, LH, AMH level, AFC, Gn dosage*, number of retrieved oocytes*, serum E2*, P level on HCG day, average size of bilateral ovaries on HCG day*, type of infertility*, average temperatures*, average minimum temperatures*, average maximum temperature*, sunshine duration. *P < 0.05.

**Figure 2:**
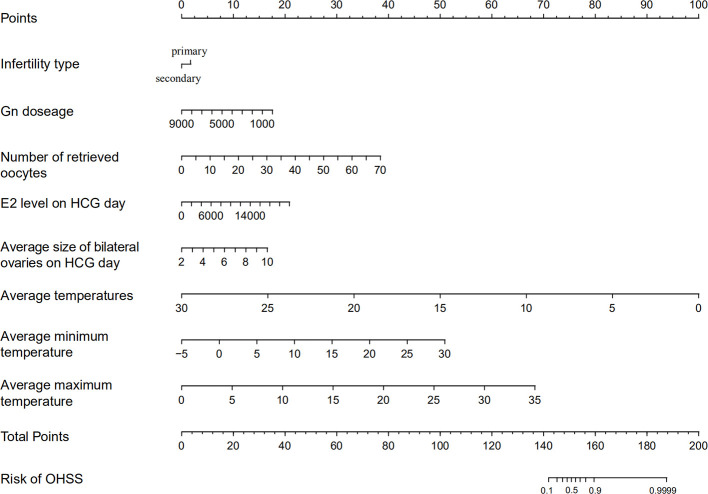
Ovarian hyperstimulation syndrome (OHSS) risk nomogram. The scaled line to the right of each variable represents the value range of the variable, while the length of the line indicates the size of the variable’s contribution to the outcome event. The value of each variable corresponds to the Points at the top of the figure, and all the scores add up to the Total points at the bottom. The risk of OHSS occurrence is represented by the Total points corresponding to the value of Risk of OHSS at the bottom of the figure.

**Figure 3:**
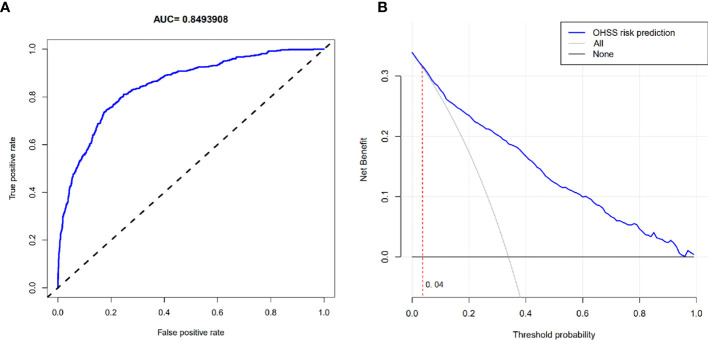
**(A)** ROC curve. **(B)** Decision curve analysis. **(B)** The horizontal solid line represents the clinical net benefit when none patients are treated after oocyte retrieval, the backslash with a negative grey slope represents the net benefit when all patients are treated. When the blue curve was above the part of the two extreme curves where the threshold probability was >4%, using this nomogram in the current study to predict OHSS risk gets more clinical benefit.

The OHSS risk prediction model that incorporated the above independent risk factors was developed and is presented as a nomogram. We performed the precision, discrimination, stability, and application value of the model by the C-index, ROC curve, bootstrap internal validation method, and DCA. We deleted sample objects with missing values under some indicators. Ultimately, 1925 individuals were included in the model analysis. The C-index was 0.849, the AUC was 0.849, and the internal validation C-index was 0.846. DCA showed that the nomogram is clinically useful when the decision to intervene at an OHSS possibility threshold of 4% (**Figures 2** and **3**).

In different seasons, the live birth rate, number of retrieved oocytes, and high-quality embryo rate were significant differences. The relationships between the above three outcomes and the seasonal factor were analyzed by linear or logistic regression; there was no correlation between the live birth rate or number of retrieved oocytes and the seasonal factor (*P* > 0.05). The high-quality embryo rate was related with sunshine duration (*P* < 0.05; **Table 3**).

**Table 3:** Association of live birth rate, number of retrieved oocytes, and high-quality embryo rate with meteorological factors.

	Live birth rate^1^			Number of retrieved oocytes^2^			High-quality embryo rate^3^		
	*P*	OR	95%CI	*P*	β	95%CI	*P*	β	95%CI
Average temperatures, °C	0.516	0.817	(0.4431.504)	0.69	0.38	(-1.151 1.751)	0.69	0.46	(-0.049 0.074)
Average minimum temperatures, °C	0.519	1.099	(0.8251.464)	0.44	-0.33	(-0.937 0.410)	0.07	-0.94	(-0.055 0.002)
Average maximum temperature, °C	0.538	1.127	(0.771.649)	0.89	-0.08	(-0.978 0.848)	0.44	0.56	(-0.023 0.054)
Sunshine duration, hr	0.714	1.001	(0.9961.006)	0.13	-0.06	(-0.021 0.003)	0.00	-0.20	(-0.002 -0.001)

Calibration variables:

^1^age*,infertility duration, infertility type, BMI*, basal serum FSH, E2, LH, AMH level, AFC, Gn dosage, number of retrieved oocytes, number of transplanted embryos, serum E2, P* level on HCG day, endometrial thickness*, average temperatures,

average minimum temperatures, average maximum temperature, sunshine hours.

^2^age, infertility duration, BMI*, basal serum FSH*, E2, LH, AMH level, AFC*, Gn dosage, serum E2*, P* level on HCG day, average size of bilateral ovaries on HCG day*, average temperatures, average minimum temperatures, average maximum temperature, sunshine duration.

^3^age, infertility duration, BMI*, basal serum FSH, E2, LH, AMH* level, AFC, Gn dosage, number of retrieved oocytes*, serum E2, P level on HCG day, average size of bilateral ovaries on HCG day, average temperatures, average minimum temperatures, average maximum temperature, sunshine duration*.

*P < 0.05.

## Discussion

OHSS is a serious, iatrogenic complication of COH. Patients with PCOS are at an increased risk for OHSS due to the high ovarian responsivity to gonadotropin stimulation (11). Our results showed that the high risk for OHSS after oocyte retrieval in PCOS patients was associated with total Gn dosage, number of retrieved oocytes, E2 level, and average diameter of the bilateral ovaries on the HCG day of administration, which was consistent with previous studies (12–15). In addition, our study showed that PCOS patients with primary infertility had a greater incidence of OHSS, which may be related to more complex symptoms.

We found that the high risk for OHSS in PCOS patients was significantly different between the seasons. Correlation analysis showed that the monthly average temperature, average maximum temperature, and average minimum temperature were independent risk factors for OHSS, suggesting that effect of extreme climates. Extreme climates (heat and/or cold) could cause vasoconstriction, the release of inflammatory factors and the activation of the local renin-angiotensin aldosterone system, all of them are associated with the development of OHSS.

Extreme temperature exposure has adverse effects on the human body, especially females and elderly people are vulnerable to the potential adverse effects (16). Winter is the season with high incidence of cardiovascular and cerebrovascular diseases because of low temperature stimulates vasocontraction. When the outdoor temperature is higher than 5°C, systolic blood pressure increases by 6.7 mmHg and diastolic blood pressure increases by 2.1 mmHg for every 10°C decrease (17). Increased blood pressure speeds up the flow of fluid from the blood vessels into the interstitial space, facilitating the onset of OHSS. In the winter, the immune system is reinforced, which in turn promotes the serum concentrations of IL-6 and IL-6R. Studies have shown that the inflammatory factor, IL-6, is involved in the pathogenesis of OHSS (8). The IL-6R/IL-6 complex acts on ovarian vascular endothelium to promote endothelial cells to secrete VEGF by activating the STAT3/ERK signaling pathway, which increases vascular permeability (18). Cold temperature also stimulates the HPA axis to secrete adrenocorticotropic hormone (ACTH) (19) and aldosterone levels increase in a dose-dependent fashion with ACTH (10). At the same time, cold temperature also affects the SNS, leading to the activation of adrenaline receptors on the juxtaglomerular cells and the release of renin, thus activating the renin-angiotensin-aldosterone system, which increases the secretion of aldosterone, causes vasoconstriction and induce OHSS (20). Similarly, heat and dryness produce anxiety, irritability, and other negative emotions that activate the HPA axis and SNS and promote aldosterone secretion (21). Studies have confirmed that four consecutive days of mice housed at 35 ± 1°C led to a significant increase in cell volume and cell count in the adrenal cortex of mice, with a 16% increase in the serum levels of aldosterone (*P* < 0.05) (22). In addition, high temperatures can cause a series of physiologic changes including body heat dissipation and activity, blood redistribution, a large amount of blood flow to the skin and muscles, and water loss in the body that can subsequently contribute to the increases and aggravation of blood concentration (23).

We subsequently constructed an OHSS risk prediction model of the independent risk factors affecting the occurrence of high risk for OHSS after oocyte retrieval in patients with PCOS, and evaluated and validated the predictive efficacy and clinical application value of this prediction model. The C-index was 0.849 and the AUC was 0.849, suggesting that the model has good precision and discrimination. The internal validation C-index was 0.846, which indicates that the prediction model is stable. The DCA revealed that the prediction model has a meaningful clinical effect when intervention was decided among nearly the entire range of threshold probabilities. The nomogram can help clinicians screen patients at high risk for OHSS and intervene as early as possible. For example, on the HCG trigger day, we can use the nomogram prediction model to screen for those at high risk for OHSS and assign a personalized trigger plan or prophylactic medication after retrieved oocytes (letrozole or cabergoline) to reduce the risk of OHSS.

Currently, the correlation between the high-quality embryo rate and season is inconsistent. The present study showed that the high-quality embryo rate was significantly lower in the spring and summer than the autumn and winter, which is consistent with the findings of Stolwik et al. (24), but in contrast to the conclusions of Rojansky et al. (25). Further studies have demonstrated that the high-quality embryo rate was correlated with sunshine duration and may be related to melatonin, which is present in follicular fluid and is involved in follicular development, ovulation, and oocyte maturation. Moreover, the high-quality embryo rate is inversely proportional to sunshine duration (26). Less sunshine in the autumn and winter leads to an increased secretion of melatonin, which has a positive effect on oocyte quality (27).

Our study first found that the incidence of PCOS patients at high risk for OHSS after oocyte retrieval in the cycle of IVF/ICSI fluctuated with temperature changes, and increased in hot or cold weather, suggesting that the dosage of Gn should be considered to reduce the risk of OHSS in climate extremes. We constructed the prediction model of OHSS risk so that clinicians can conduct personalized and effective prevention and treatment measures for patients to reduce the risk of OHSS. Our study was limited by region and sample size. In the future, we will conduct a multi-center and large-sample size study to further explore the factors related to a high risk for OHSS and verify the predictive efficacy and clinical application value of the predictive model.

## Data Availability Statement

The raw data supporting the conclusions of this article will be made available by the authors, without undue reservation.

## Ethics Statement

The studies involving human participants were reviewed and approved by Ethics Committee for scientific research and clinical trials of the First Affiliated Hospital of Zhengzhou University. Written informed consent for participation was not required for this study in accordance with the national legislation and the institutional requirements.

## Author Contributions

JZ contributed to the conception of study. YC, HS, and YM contributed to design work. YC was responsible for statistical analyses performing and manuscript writing. LM contributed to revising the manuscript. All authors contributed to the article and approved the submitted version.

## Funding

The study was supported by the National Science Foundation of China under Grant to JZ (82071649).

## Conflict of Interest

The authors declare that the research was conducted in the absence of any commercial or financial relationships that could be construed as a potential conflict of interest.
